# Antioxidant and Antiproliferative Activities of Heterofucans from the Seaweed *Sargassum filipendula*

**DOI:** 10.3390/md9060952

**Published:** 2011-06-07

**Authors:** Leandro Silva Costa, Gabriel Pereira Fidelis, Cinthia Beatrice Silva Telles, Nednaldo Dantas-Santos, Rafael Barros Gomes Camara, Sara Lima Cordeiro, Mariana Santana Santos Pereira Costa, Jailma Almeida-Lima, Raniere Fagundes Melo-Silveira, Ruth Medeiros Oliveira, Ivan Rui Lopes Albuquerque, Giulianna Paiva Viana Andrade, Hugo Alexandre Oliveira Rocha

**Affiliations:** 1 Laboratório de Biotecnologia de Polímeros Naturais (BIOPOL), Departamento de Bioquímica, Centro de Biociências, Universidade Federal do Rio Grande do Norte (UFRN), Natal, Rio Grande do Norte, Brazil; E-Mails: gabrielfideliss@gmail.com (G.P.F.); cinthiatelles@yahoo.com.br (C.B.S.T), nednaldod@hotmail.com (N.D.-S); rafael_bgc@yahoo.com.br (R.B.G.C.); sara-cordeiro@hotmail.com (S.L.C.); marispc_bio@yahoo.com.br (M.S.S.P.C.); biolottus23@yahoo.com.br (J.A.-L); ranierefagundes@hotmail.com (R.F.M.-S); rmo_85@hotmail.com (R.M.O.); ivan.rui@click21.com.br (I.R.L.A); giulipaiva@cb.ufrn.br (G.P.V.A.); 2 Federal Institute of Education, Science and Technology of Rio Grande do Norte (IFRN), Santa Cruz, Rio Grande do Norte, Brazil; E-Mail: leandro-silva-costa@hotmail.com (L.S.C.)

**Keywords:** fucoidan, biological activities, HeLa cells, brown seaweed

## Abstract

Fucan is a term used to denominate a type of polysaccharide which contains substantial percentages of l-fucose and sulfate ester groups. We obtained five heterofucans from *Sargassum filipendula* by proteolytic digestion followed by sequential acetone precipitation. These heterofucans are composed mainly of fucose, glucose, glucuronic acid, galactose and sulfate. These fucans did not show anticoagulant activity in PT and aPTT tests. Their antioxidant activity was evaluated using the follow tests; total antioxidant capacity, scavenging hydroxyl and superoxide radicals, reducing power and ferrous ion [Fe(II)] chelating. All heterofucans displayed considerable activity, especially SF-1.0v which showed the most significant antioxidant potential with 90.7 ascorbic acid equivalents in a total antioxidant capacity test and similar activity when compared with vitamin C in a reducing power assay. The fucan antiproliferative activity was performed with HeLa, PC3 and HepG2 cells using MTT test. In all tested conditions the heterofucans exhibited a dose-dependent effect. The strongest inhibition was observed in HeLa cells, where SF-1.0 and SF-1.5 exhibited considerable activity with an IC50 value of 15.69 and 13.83 μM, respectively. These results clearly indicate the beneficial effect of *S. filipendula* polysaccharides as antiproliferative and antioxidant. Further purification steps and additional studies on structural features as well as *in vivo* experiments are needed to test the viability of their use as therapeutic agents.

## 1. Introduction

Sulfated polysaccharides are a complex group of macromolecules with a wide range of important biological properties. Marine algae are the most important source of non-animal sulfated polysaccharides. These biomolecules are widely studied owing to their broad therapeutic applications such as antithrombotic, anticoagulant, antioxidant, anti-inflammatory and antiproliferative compounds [1–4].

In recent years, several groups have reported that sulfated polysaccharides obtained from diverse species of the genus Sargassum exhibit various biological activities: *Sargassum horneri* [5]; *S. tenerrimum* [6]; *S. patens* [7]; *S. stenophyllum* [8] and *S. wightii* [9]. More recently, our group obtained a sulfated polysaccharide-rich extract from *Sargassum filipendula*, common seaweed along the northeast coast of Brazil. It exhibited antiproliferative and antioxidant activities [2]. However, biological activities of purified sulfated polysaccharides from *S. filipendula* have not been examined.

In this context, the aim of this study was to obtain sulfated polysaccharides from *S. filipendula* and to evaluate their biological activities, including anticoagulant, antioxidant, and antiproliferative. Our results show several noteworthy differences in the activities of polysaccharides from *S. filipendula*, likely connected to differences in the chemical structure of these compounds. This work focused on the selection of the most active sulfated polysaccharide samples for further study as potential novel drugs for thrombosis, antioxidant and/or antitumor therapy. The sulfated polysaccharide denominated SF-1.5v was specifically selected to determine its possible antiproliferative mechanism in future studies.

## 2. Results and Discussion

### 2.1. Chemical Analyses

In this study, using methodology that combined proteolysis and acetone precipitation, we obtained five polysaccharides from the brown seaweed *S. filipendula* denominated: SF-0.5v, SF-0.7v, SF-1.0v, SF-1.5v, and SF-2.0v. Chemical analysis of sulfated polysaccharides is summarized in Table 1. The monosaccharide composition of sulfated polysaccharides is also shown in Table 1. Fucose, galactose, glucose, mannose, xylose, and glucuronic acid were found in different amounts in each polysaccharide. Exceptions were mannose and glucuronic acid, which were not detected in SF-1.5 and SF-2.0v, respectively. Data showed that galactose and fucose were the main sugars present in all the polysaccharides, indicating that we obtained five different types of sulfated galactofucans. Since the 1950s [10], several sulfated fucans containing xylose and galactose have been described, but only a few with galactose as the major component have been described in seaweed: *Saccharina longicruris* [11], *Lobophora variegata* [12], *Spatoglossum schroederi* [10], *Adenocystis utricularis* [13], and *Sargassum stenophyllum* [14]. Accordingly, we sought to analyze the chemical, anticoagulant, antioxidant, and antiproliferative properties of these heterofucans from *S. filipendula*.

The amount of total sugar and sulfate increased in polysaccharides obtained with high acetone volumes. The heterofucan SF-2.0v showed the highest total sugar content (66.0%) and sulfate content (17.7%) when compared with other sulfated polysaccharides. On the other hand, SF-0.5v and SF-0.7v exhibited the lowest total sugar content (41.4% and 46.2%, respectively) and sulfate content (10.2% and 10.8%, respectively). Additionally, all the polysaccharides showed low protein contamination, ranging from 0.2 (SF-1.5v) to 0.6% (SF-1.5v). Considering total sugar, sulfate and protein content, total percentage varies from 52.2% to 84.1% in SF-0.5 and SF-2.0, respectively. Most articles that show the proportion of proteins, carbohydrates and heterofucan sulfate exhibit data in the same fashion as we present ours [6,15,16]. The sum of the three components found in the heterofucans of these articles does not approach 100%. This is due to the fact that these polymers are very hygroscopic, absorbing water from the atmosphere very rapidly after lyophilization. Furthermore, because of the negative loads of sulfate clusters and glucoronic acids, metals are not eliminated from fucan structures, even after dialysis. Another important point is the coformation that these polymers exhibit in aqueous solutions, which may capture cations within their structures.

### 2.2. Anticoagulant Activity by Activated Partial Thromboplastin Time (aPTT) and Prothrombin Time (PT) Assays

Fucans have a wide variety of biological activities, but their potent anticoagulant action is by far the most widely studied. The anticoagulant activity of heterofucans from *S. filipendula* was evaluated by PT and aPTT tests to assess the extrinsic and intrinsic pathway of coagulation, respectively. In the present study, none of the sulfated polysaccharides showed anticoagulant activity in any of the tested conditions. A similar observation was reported for a range of sulfated galactans from marine invertebrates and heterofucans from brown seaweeds [3,17]. This result suggests that the anticoagulant effect of fucans is stereospecific and not merely a consequence of their charge density or sulfate content [18]. The position of sulfate groups on sugar residues is also very important for the anticoagulant activity of fucans. Thus, sulfated polysaccharides extracted from S*. filipendula* likely do not have a favorable structure for interacting with proteins involved in the clotting process and therefore do not exhibit anticoagulant activity.

### 2.3. Antioxidant Activity

Several sulfated polysaccharides from marine algae have been described as having antioxidant activity [19–22]. Thus, sargassum heterofucan antioxidant activities were evaluated in different antioxidant assays: total antioxidant capacity (TAC), scavenging hydroxyl and superoxide radicals, power reducing and ferrous chelating.

#### 2.3.1. Total Antioxidant Capacity (TAC)

**Figure 1:**
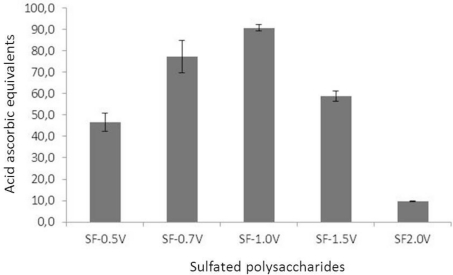
Total antioxidant capacity of sulfated polysaccharides from *Sargassum filipendula.* Each value is the mean ± SD of five determinations. All the sulfated polysaccharides showed a significant difference (p < 0.05).

The TAC assay allows determination the antioxidant potential of natural compounds. All sulfated polysaccharides from *S. filipendula* exhibited activity in TAC assay (expressed as ascorbic acid equivalents). All heterofucans also showed activity (Figure 1). SF-2.0v had the lowest activity, with 9.6 ascorbic acid equivalents. The other fucans exhibited considerable antioxidant activity, especially SF-0.7v and SF1.0v, with 77.3 and 90.7 ascorbic acid equivalents, respectively. Thus, Sargassum heterofucans can be separated into three groups: Low (SF-2.0v), intermediate (SF-0.5v and SF-1.5v) and high (SF-0.7v and SF1.0v) antioxidant potential. Additionally, SF-0.5v, SF-0.7v, SF1.0v, and SF-1.5v showed higher TAC than other fucans such as those purified from *Padina tetrastomatica*, *Turbinaria conoides* and *Canistrocarpus cervicornis* [23].

#### 2.3.2. Hydroxyl and Superoxide Radical Scavenging

Table 2 depicts the results obtained for the inhibition of hydroxyl radicals and superoxide anion formation. Only sulfated polysaccharides SF-0.7v, SF-1.0v, and SF-1.5v showed activity in hydroxyl radical scavenging in a dose dependent manner. However, these polysaccharides exhibited moderate scavenging activity of 23.0%, 26.7%, and 12.7%, respectively, at 0.5 mg/mL. For this assay, gallic acid showed 93.7% radical scavenging effect at 0.5 mg/mL. In the superoxide anion scavenging assay, fucans SF-0.5v, SF-1.0v, and SF-1.5v did not show antioxidant activity, while SF-0.7v and SF2.0v displayed moderate superoxide scavenging with 19.3 and 16.9% at 0.5 mg/mL, respectively.

In an earlier study [2], a sulfated polysaccharide-rich extract from *S. filipendula* showed no activity in superoxide and hydroxyl scavenging assays. Interestingly, in this study all sulfated polysaccharides purified from *S. filipendula*, with the exception of SF-0.5v (Table 2), were able to scavenge these free radicals.

The literature has systematically reported several sulfated polysaccharides extracted from algae without hydroxyl and superoxide radical scavenging activity [2,24]. These data show that hydroxyl and superoxide radical scavenging is probably not the major antioxidant mechanism of these heterofucans.

#### 2.3.3. Chelating Effect on Ferrous Ions

**Figure 2:**
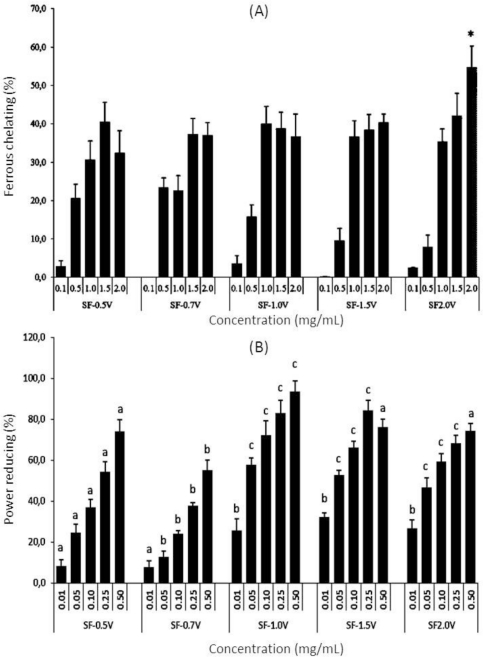
Antioxidant activity of sulfated polysaccharides from *Sargassum filipendula*. (**A**) Ferric chelating assay; (**B**) Power reducing assay. Each value is the mean ± SD of five determinations. * The only sulfated polysaccharide to show different sulfated polysaccharides when compared at each concentration. ^a,b,c,d^ Different letters indicate a significant difference between sulfated polysaccharides when compared at each concentration.

Hydroxyl radicals are the most aggressive free radicals; however, the scavenger of these radicals *in vivo* is not very effective. The main mechanism suppresses the generation of hydroxyl radicals. When the antioxidant links to the metal ions, it impedes the latter from interacting with H_2_O_2_, in turn impeding H_2_O_2_ from decomposing, and forms a stronger free radical. The metal complexes thus formed cannot further react with H_2_O_2_ to produce a hydroxyl radical. In this work, all sulfated polysaccharides from *S. filipendula* showed excellent ferrous ion [Fe(II)] chelating capacity. These are great results, since ferrous ions are considered to be the most effective pro-oxidants present in food systems. The plot of iron-chelating capacity as a function of sample concentration is shown in Figure 2A.

The results revealed that all heterofucans exhibit significant dose-dependent ferrous chelating capacity. With the exception of SF-2.0v, all sulfated polysaccharides showed similar iron-chelating capacity at tested concentrations (p < 0.05). Additionally, higher SF-2.0v activity (p < 0.05) can be observed at elevated concentrations (2.0 mg/mL), with 54.8% ferrous chelating. This activity was only 1.7 times lower than EDTA (positive control) activity at the same concentration under the same experimental condition (Data not shown).

The sulfated polysaccharide-rich extract from *S. filipendula* showed low ferric chelating activity (13.2% chelation at 2.0 mg/mL) [2]. As observed in hydroxyl and superoxide radical scavenging assays, heterofucans from *S. filipendula* showed better activity than sulfated polysaccharide-rich extract from *S. filipendula.* These data allow us to conclude that the antioxidant activity of sulfated polysaccharide-rich extract from *S. filipendula* is due to the sulfated polysaccharides present.

#### 2.3.4. Reducing Power

The reducing power assay was expressed as percentage activity of ascorbic acid control at 0.1 mg/mL (Figure 2B). The heterofucan SF-0.7v showed only moderate activity when compared with that of vitamin C at 0.1 mg/mL. Furthermore, fucans SF-1.0v and SF-1.5v showed considerable reducing power, especially SF-1.0v, which, at a high concentration (0.5 mg/mL), showed activity similar to that found for vitamin C.

### 2.4. Antiproliferative Activity

**Figure 3:**
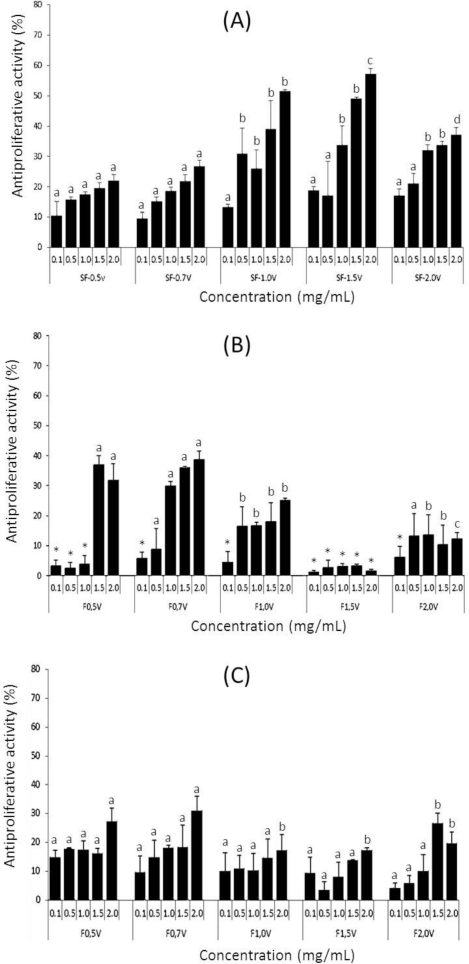
Influence of sulfated polysaccharides from *Sargassum filipendula* on cell proliferation inhibition of tumor cells after 24 h incubation. (**A**) antiproliferative activity in HeLa (Human cervical cancer cell lines); (**B**) antiproliferative activity in HepG2 (Human hepatocellular carcinom cell lines); (**C**) antiproliferative activity in PC3 (human prostate cancer cell lines). Each value is the mean ± SD of seven determinations. Different letters indicates a significant difference between concentrations of individual sulfated polysaccharides (p < 0.05). * antiproliferative activity not detected.

Antiproliferative activity was performed with HeLa, PC3 and HepG2 cells under the following conditions: 24h of incubation at 0.1, 0.5, 1.0, 1.5 and 2.0 mg/mL (Figure 3). In all tested conditions sulfated polysaccharides from *Sargassum filipendula* exhibited a dose-dependent effect. The only exception was SF-1.5v, which showed no inhibition to HepG2 cells. PC3 and HepG2 cells were only moderately inhibited by the polysaccharides analyzed. SF-0.7v was the most effective polysaccharide for these cells, inhibiting HepG2 and PC3 by 38.1% and 31.0%, respectively.

The strongest inhibition of sulfated polysaccharides from *S. filipendula* was observed in HeLa cells. In this experiment, polysaccharides SF-0.5v, SF-0.7v and SF-2.0v can be classified as a moderate activity group when compared with SF-1.0v and SF-1.5v, which are classified as a high activity group. Sulfated polysaccharides from the moderate activity group showed maximum antiproliferative activity, with 37.1% inhibition at 2.0 mg/mL (SF-2.0v). However, it was not possible to determine the IC_50_ value for these polysaccharides. By contrast, sulfated polysaccharides from the high activity group showed significant activity and IC_50_ value of 15.69 μM (SF-1.0v) and 13.83 μM (SF-1.5v). However, this IC_50_ values are not comparable to those of positive controls, doxorubicin (IC_50_ 6.8 μM) and 5-fluorouracil (IC_50_ 8.0 μM). On the other hand, the sargassum heterofucans exhibited more potent antiproliferative activity against tumor cells in comparison to heterofucans from other source, like as heterofucan from *Cladosiphon okamuranus* that inhibits HepG2 proliferation showing IC value of 50 μM [25], and a heterofucan from *Ascophyllum nodosum* that inhibits HeLa cells proliferation, but this IC_50_ value was not determinate even using high amounts of polysaccharide [26].

Based on the experiment carried out in the present study, it is clear that the effect of fucans on different cell lines is unequal and therefore there must be a preference for certain cells, that is, the effect of the fucan is cell dependent. A number of *in vivo* and *in vitro* studies on the antitumor activity of fucans have been conducted [27,28]. Although the precise mechanisms underlying this activity remain to be determined, a few possibilities have been proposed. It has been speculated that fucans act by inhibiting tumor angiogenesis, modulating host immune systems [27], arresting the cell cycle and/or inducing apoptosis [29]. Thus, further studies are needed to clarify the antiproliferative mechanism of fucans from *S. filipendula*, especially SF-1.5v, which exhibited the strongest activity.

## 3. Experimental Section

### 3.1. Materials

Iron (II) sulfate, potassium ferricianyde, sulfuric acid and acetonitrile were obtained from Merck (Darmstadt, Germany). Nitro Blue Tetrazolium (NBT), monosaccharides, doxorubicin, 5-fluorouracil, methionine and ammonium molybdate were purchased from Sigma-Aldrich Co. (St. Louis, USA). All other solvents and chemicals were of analytical grade.

### 3.2. Extraction of Sulfated Polysaccharide

The Phaeophyta *Sargassum filipendula* was collected at Búzios Beach, Nísia Floresta, Brazil. The alga was stored in our laboratory and dried at 50 °C under ventilation in an oven, ground in a blender and incubated with acetone to eliminate lipids and pigments. About 100 g of powdered alga was suspended with five volumes of 0.25 M NaCl and the pH was adjusted to 8.0 with NaOH. Next, 900 mg of Prolav 750 (Prozyn Biosolutions, São Paulo, SP, Brazil), a mixture of alkaline proteases, was added for proteolytic digestion. After incubation for 24 h at 60 °C under agitation and periodical pH adjustments, the mixture was filtered through cheesecloth. The filtrate was fractionated by precipitation with acetone as follows: 0.5 volumes of ice-cold acetone was added to the solution under gentle agitation and maintained at 4 °C for 24 h. The precipitate formed was collected by centrifugation (10000 × g, 20 min), vacuum dried, resuspended in distilled water, and analyzed. The operation was repeated by adding 0.7, 1.0, 1.5 and 2.0 volumes of acetone to the supernatant.

### 3.3. Chemical Analysis and Monosaccharide Composition

Total sugars were estimated by the phenol-H_2_SO_4_ reaction [30] using l-fucose as standard. Sulfate content was determined according to the gelatin-barium method [31], using sodium sulfate (1 mg/mL) as standard and after acid hydrolysis of the polysaccharides (4 M HCl, 100 °C, 6 h). Protein content was measured using Spector’s method [32].

The polysaccharides were hydrolyzed with 0.5, 1, 2, and 4 M, respectively, for various lengths of time, (0.5, 1, 2 and 4 h), at 100 °C. Reducing sugars were determined using the Somogyi-Nelson method [33]. After acid hydrolysis, sugar composition was determined by a LaChrom Elite^®^ HPLC system from VWR-Hitachi with a refractive index detector (RI detector model L-2490). A LichroCART^®^ 250-4 column (250 mm × 40 mm) packed with Lichrospher^®^ 100 NH_2_ (5 μm) was coupled to the system. The sample mass used was 0.2 mg and analysis time was 25 minutes. The following sugars were analyzed as references: arabinose, fructose, fucose, galactose, glucose, glucosamine, glucuronic acid, mannose, and xylose.

### 3.4. Molecular Weight Determination

The sulfated polysaccharides were subjected to gel-permeation chromatography on Sephadex G-100 (140 × 1 cm) using 0.2 M acetic acid/0.15M NaCl as eluent. The elution was monitored for total sugar [30] and metachromasia [34]. To estimate the molecular weight of the polysaccharides, dextrans of different molecular weights were used as standards.

### 3.5. Anticoagulant Activity

Prothrombin time (PT) and activated partial thromboplastin time (aPTT) coagulation assays were performed with a coagulometer as described earlier [35] and measured using normal citrate-treated human plasma. All assays were performed in duplicate and repeated at least three times on different days (n = 6). The results were expressed as aPTT ratio, which was determined as follows: aPTT control time/aPTT sample time.

### 3.6. Antioxidant Activity

Five assays were performed to analyze the antioxidant activity of the sulfated polysaccharides obtained: total antioxidant capacity, hydroxyl radical scavenging, superoxide radical scavenging, ferric chelating and reducing power, as previously described [2].

#### 3.6.1. Determination of Total Antioxidant Capacity

This assay is based on the reduction of Mo (VI) to Mo (V) by sulfated polysaccharides and subsequent formation of a green phosphate/Mo(V) complex at acid pH. Tubes containing sulfated polysaccharides and reagent solution (0.6 M sulfuric acid, 28 mM sodium phosphate and 4 mM ammonium molybdate) were incubated at 95 °C for 90 min. After the mixture had cooled to room temperature, the absorbance of each solution was measured at 695 nm against a blank. Total antioxidant capacity was expressed as ascorbic acid equivalent.

#### 3.6.2. Hydroxyl Radical Scavenging Activity Assay

The scavenging activity of seaweed polysaccharides against the hydroxyl radical was investigated using Fenton’s reaction (Fe^2+^ + H_2_O_2_→Fe^3+^ + OH^−^ + OH^•^). These results were expressed as inhibition rate. Hydroxyl radicals were generated using 3 mL sodium phosphate buffer (150 mM, pH 7.4), which contained 10 mM FeSO_4_.7H_2_O, 10 mM EDTA, 2 mM sodium salicylate, 30% H_2_O_2_ (200 mL) and varying polysaccharide concentrations. In the control, sodium phosphate buffer replaced H_2_O_2_. The solutions were incubated at 37 °C for 1 h, and the presence of the hydroxyl radical was detected by monitoring absorbance at 510 nm. Gallic acid was used as positive control.

#### 3.6.3. Superoxide Radical Scavenging Activity Assay

This assay was based on the capacity of sulfated polysaccharides to inhibit the photochemical reduction of nitroblue tetrazolium (NBT) in the riboflavin–light–NBT system. Each 3 mL of reaction mixture contained 50 mM phosphate buffer (pH 7.8), 13 mM methionine, 2 mM riboflavin, 100 mM EDTA, NBT (75 mM) and 1 mL sample solution. After the production of blue formazan the increase in absorbance at 560 nm after 10 min illumination from a fluorescent lamp was determined. The entire reaction assembly was enclosed in a box lined with aluminum foil. Identical tubes with the reaction mixture were kept in the dark and served as blanks. Gallic acid was used as positive control.

#### 3.6.4. Ferrous Ion [Fe(II)] Chelating Activity

The ferrous ion chelating ability of samples was investigated using the following methodology: sulfated polysaccharides at different concentrations were applied with the reaction mixture, which contained FeCl_2_ (0.05 mL, 2 mM) and ferrozine (0.2 mL, 5 mM). The mixture was shaken and incubated for 10 min at room temperature and absorbance of the mixture was measured at 562 nm against a blank. EDTA was used as positive control.

#### 3.6.5. Reducing Power

The reducing power of the samples was quantified as described later [36]. Briefly, 4 mL of reaction mixture, containing different sample concentration in phosphate buffer (0.2 M, pH 6.6), was incubated with potassium ferricyanide (1% w/v) at 50 ºC for 20 min. The reaction was stopped by TCA solution (10% w/v). The solution was then mixed with distilled water and ferric chloride (0.1% w/v) solution and the absorbance was measured at 700 nm. The result was expressed as a percentage of the activity shown by 0.2 mg/mL of Vitamin C.

### 3.7. Antiproliferative Activity

The cells culture (HeLa, HepG2 and PC3) were grown in 75 cm^2^ flasks in DMEM medium. Cells were seeded into 96-well plates at a density of 5 × 10^3^ cell/well and allowed to attach overnight in 300 μL medium incubated at 37 ºC, 5% CO_2_. The sulfated polysaccharides fractions were added at a final concentration of 0.1; 0.5; 1.0; 1.5 and 2.0 mg/mL, for 24 h at 37 ºC and 5% CO_2_. After incubation, traces of sulfated polysaccharides fractions were removed by washing the cells twice with 200 μL PBS and applying 100 μL of fresh medium plus and 10 μL of 12 mM MTT dissolved in PBS to determine the effects of the algal sulfated polysaccharides on cell proliferation. Cells were then incubated for 4 h at 37 ºC, 5% CO_2_. To solubilize the product of MTT cleavage, 100 μL of isopropanol containing 0.04 N HCl was added to each well and thoroughly mixed using a multichannel pipettor. Within 1 h of HCl-isopropanol addition, the absorbance at 570 nm was read using a Multiskan Ascent Microplate Reader (Thermo Labsystems, Franklin, MA, USA). The percent inhibition of cell proliferation was calculated as follows:

$$\%\;\text{Inhibition}=\frac{\text{Abs}.\;570\;\text{nm Control}-\text{Abs}.\;570\;\text{nm}\;\text{sample}}{\text{Abs}.\;570\;\text{nm Control}}\times 100$$

### 3.8. Statistical Analysis

All data were expressed as mean ± standard deviation. Statistical analysis was done by one-way Anova using the SIGMAStat 2.01 software. Student-Newmans-Keuls post-tests were performed for multiple group comparison. In all cases statistical significance was set at p < 0.05.

## 4. Conclusions

In conclusion, we extracted five heterofucans from the brown seaweed *S. filipendula*, showing antioxidant and antiproliferative activities at different levels. This study focused on the selection of the most active sulfated polysaccharide samples for further study as potential novel drugs for antioxidant and/or antitumor therapy. SF-1.5v was specifically selected to determine its possible antiproliferative mechanism in future studies.

## Figures and Tables

**Table 1:** Chemical composition of sulfated polysaccharides obtained from *Sargassum filipendula*.

**Sulfated Polysaccharides**	**Total sugar (%)**	**Sulfate (%)**	**Protein (%)**	**Molar ratio**					
				**Fuc**	**Gal**	**Glc**	**Man**	**Xyl**	**Gluc acid**
**SF-0.5v**	41.4	10.2	0.6	1.0	1.5	0.5	0.4	1.0	1.1
**SF-0.7v**	46.2	10.8	0.5	1.0	1.2	0.7	0.2	0.7	0.7
**SF-1.0v**	59.1	12.6	0.3	1.0	1.3	0.5	0.1	0.3	0.7
**SF-1.5v**	64.9	12.3	0.2	1.0	1.1	0.3	-	0.1	0.5
**SF-2.0v**	66.0	17.7	0.4	1.0	2.2	0.5	0.6	0.2	-

Fuc: fucose; Gluc acid: glucuronic acid; Gal: galactose; Xyl: xylose; Man: mannose; Glc: glucose; -: Traces; n.d: not detected.

**Table 2:** Hydroxyl and Superoxide radical scavenging activity of sulfated polyssacharides from *Sargassum filipendula.*

**Sulfated polysaccharides**	**Concentration (mg/mL)**	**Scavenging (%)**	
		**OH^•^**	**O_2_^−^**
SF-0.5v	0.05	0 ± 0	0 ± 0
	0.10	0 ± 0	0 ± 0
	0.25	0 ± 0	0 ± 0
	0.50	0 ± 0	0 ± 0
SF-0.7v	0.05	5.3 ± 3.5 ^a^	11.1 ± 0.4 ^a^
	0.10	15.8 ± 2.9 ^b^	14.2 ± 0.4 ^b^
	0.25	23.0 ± 1.5 ^c^	16.3 ± 0.6 ^b^
	0.50	26.2 ± 1.8 ^c^	19.3 ± 0.7 ^b^
SF-1.0v	0.05	10.8 ± 2.2 ^a^	0 ± 0
	0.10	17.1 ± 2.9 ^b^	0 ± 0
	0.25	22.5 ± 1.5 ^c^	0 ± 0
	0.50	26.7 ± 1.8 ^c^	0 ± 0
SF-1.5v	0.05	4.9 ± 0.9 ^a^	0 ± 0
	0.10	9.2 ± 0.7 ^b^	0 ± 0
	0.25	12.4 ± 1.5 ^b^	0 ± 0
	0.50	12.7 ± 4.8 ^b^	0 ± 0
SF-2.0v	0.05	0 ± 0	0 ±0
	0.10	0 ± 0	5.0 ± 0.7 ^a^
	0.25	0 ± 0	9.0 ± 1.1 ^b^
	0.50	0 ± 0	12.2 ± 1.2 ^b^
Gallic acid	0.05	11.6 ± 1,7 ^a^	28.9 ± 3.8 ^a^
	0.10	43.6 ± 2.4 ^b^	41.8 ± 4.7 ^b^
	0.25	64.3 ± 3.0 ^c^	72.1 ± 2.9 ^c^
	0.50	93.7 ± 3.7 ^d^	86.3 ± 3.1 ^d^

Each value is the mean ± SD of three determinations.

a,b,c,d Different letters indicate a significant difference (p < 0.05) between each concentration of the same sulfated polysaccharide.
